# Neurovascular and neurometabolic derailment in aging and Alzheimer's disease

**DOI:** 10.3389/fnagi.2015.00103

**Published:** 2015-05-27

**Authors:** Cátia F. Lourenço, Ana Ledo, Cândida Dias, Rui M. Barbosa, João Laranjinha

**Affiliations:** ^1^Center for Neuroscience and Cell Biology, University of CoimbraCoimbra, Portugal; ^2^Faculty of Pharmacy, University of CoimbraCoimbra, Portugal

**Keywords:** neurovascular coupling, neurometabolism, nitric oxide, Alzheimer's disease, aging

## Abstract

The functional and structural integrity of the brain requires local adjustment of blood flow and regulated delivery of metabolic substrates to meet the metabolic demands imposed by neuronal activation. This process—neurovascular coupling—and ensued alterations of glucose and oxygen metabolism—neurometabolic coupling—are accomplished by concerted communication between neural and vascular cells. Evidence suggests that neuronal-derived nitric oxide (^•^NO) is a key player in both phenomena. Alterations in the mechanisms underlying the intimate communication between neural cells and vessels ultimately lead to neuronal dysfunction. Both neurovascular and neurometabolic coupling are perturbed during brain aging and in age-related neuropathologies in close association with cognitive decline. However, despite decades of intense investigation, many aspects remain poorly understood, such as the impact of these alterations. In this review, we address neurovascular and neurometabolic derailment in aging and Alzheimer's disease (AD), discussing its significance in connection with ^•^NO-related pathways.

## Introduction

The proper function of the brain depends critically upon constant and regulated blood supply. Despite representing only 2% of total body mass in the adult human, the brain is an energy expensive organ, consuming *circa* one fifth of the available oxygen and glucose (Zlokovic, 2011). Upon neural excitation, local metabolic rate may increase as much as 50% relative to basal values depending on the intensity of stimulation (Shulman and Rothman, 1998). Paradoxically, the intrinsic energy reserves are minimal (Kealy et al., 2013), which implies that, to assure an appropriate balance, changes in blood supply must be attuned to the physiological demands imposed by neural activation with high temporal and regional precision. The accomplishment of such interplay depends on the complex and concerted communication between neurons, astrocytes, pericytes, microglia, and vascular cells. Active neurons generate signals that are transduced at blood vessels to locally adjust blood flow and guarantee efficient delivery of bioenergetic substrates—a process termed neurovascular coupling. Furthermore, the profile of neuronal activity is closely associated with glucose and oxygen metabolism—neurometabolic coupling.

Neurovascular coupling has been a matter of intense investigation over the last decades and is yet not fully understood. Nonetheless, there are generally accepted propositions, such as (1) the process relies on glutamate-dependent pathways, in a feed-forward mechanism; (2) likely several molecules and/or pathways cooperate to translate the need for substrates imposed by the neuronal activity into changes in cerebral blood flow, and (3) the underlying mechanisms can be distinct throughout the brain areas, reflecting specificities of the neuronal networks. Amongst several molecules proposed to mediate neurovascular coupling, nitric oxide (^•^NO), a free radical intercellular messenger, has emerged as an attractive candidate (Iadecola, 1993). In the brain, ^•^NO is produced upon glutamatergic activation by the neuronal isoform of nitric oxide synthase (nNOS), which is physically anchored and functionally coupled to the NMDA-type glutamate receptor (Christopherson et al., 1999). Nitric oxide is endowed with peculiar physicochemical properties (radical nature, small size, diffusibility, hydrophobicity), that determine a diversity of biological targets and pathways in which it is involved (for review Winkler and Luer, 1998; Guix et al., 2005).

Despite some inconsistent observations, ample evidence suggests that ^•^NO plays a critical role in neurovascular coupling, particularly in the hippocampus and cerebellum (Rancillac et al., 2006; Lourenço et al., 2014a). Direct and *in vivo* data show that ^•^NO is a direct mediator of the process, bridging neurons and blood vessels. The simultaneous monitoring of ^•^NO fluctuations and CBF changes during glutamatergic activation, coupled to pharmacological approaches, strengthens the notion that ^•^NO produced by neurons can diffuse toward neighboring blood vessels and promote vasodilation via activation of soluble guanylate cyclase (sGC). In turn, the involvement of endothelial-derived ^•^NO appears to be negligible (Lourenço et al., 2014a). In addition to participating in neuron-to-blood vessel signaling pathways, ^•^NO may be involved in the regulation of ensued processes, such as neurometabolic coupling. Nitric oxide can regulate energy metabolism/cellular respiration by interfering with several signaling pathways. For instance, at low nM concentrations ^•^NO regulates mitochondrial respiration by inhibiting cytochrome c oxidase (CcO) in competition with O_2_ (Rossignol et al., 2003; Moncada and Bolaños, 2006; Antunes and Cadenas, 2007). This competitive process allows not only for the fine tuning of mitochondrial respiration, but may also facilitate O_2_ distribution from the microvasculature to sites of up-regulated energetic demand (Giulivi, 2003; Victor et al., 2009) or modulate production of mitochondrial-derived signaling molecules such as superoxide and hydrogen peroxide (Cadenas, 2004). Nonetheless, when ^•^NO fluxes are increased in biological systems in concurrence with an unbalanced redox environment, production of ^•^NO-derived reactive species such as peroxynitrite can significantly perturb mitochondrial function by inhibiting complex I of the mitochondrial respiratory chain, aconitase and Mn-superoxide dismutase (Brown, 2007). This Dr. Jekyll and Mr. Hyde type of bioactivity can also be observed for glycolysis, where ^•^NO has been shown to boost glycolytic turnover in a cGMP dependent mechanism in astrocytes (but not neurons) (Bolanos et al., 2008) while gluteraldehyde-3-phosphate dehydrogenase can be inhibited by nitration (Palamalai and Miyagi, 2010).

Interestingly, the impact of ^•^NO on brain metabolic status can reflect upon neurovascular coupling. Variations in O_2_ concentration can alter vascular tone, both by affecting the synthesis of the vasoactive messengers (including ^•^NO itself), and by altering the levels of lactate and adenosine, which modulate pathways underlying neurovascular coupling (Gordon et al., 2008; Attwell et al., 2010).

In sum, neuronal activity produces ^•^NO, a messenger that diffuses to blood vessels inducing vasodilation. Consequently, increased delivery of energy substrates impacts local neuronal metabolism and function. Therefore, the two processes, neuronal activity-dependent CBF increase and oxygen and glucose utilization by active neural cells are inextricably linked, establishing a functional metabolic axis in brain, the neurovascular-neuroenergetic coupling axis. Nitric oxide is a master regulator of this axis. The perturbation of this ^•^NO-driven regulatory cycle can trigger a sequence of events that may ultimately lead to neuronal dysfunction. Ample evidence supports the notion that alterations of the regulatory mechanisms involved in neurovascular and neurometabolic coupling lead to neuronal dysfunction and disease, as discussed in the following sections.

## Dysfunction in neurovascular coupling during brain aging and Alzheimer's disease

It is increasingly accepted that brain aging and age-associated diseases, such as Alzheimer's disease (AD), share some common histological and pathophysiological alterations that, ultimately, underlie compromised cognitive status. The possible contribution of abnormal cerebrovascular function to progressive functional decline has been vigorously emphasized and is nowadays well recognized. However, the putative triggers of this dysfunction remain a matter of extensive debate (Zlokovic, 2011; Kalaria, 2012; de la Torre, 2012). Age is the most relevant risk factor for the sporadic form of AD, the leading cause of dementia in the elderly. Although AD results predominantly from neurodegenerative changes, there is a growingly recognized contribution of well-defined decline in cerebrovascular parameters (Girouard and Iadecola, 2006). Between 60 and 90% of AD patients exhibit cerebrovascular pathologies including cerebral amyloid angiopathy, microinfarcts and ischemic lesions, blood–brain barrier disruption, and microvascular degeneration (Jellinger and Mitter-Ferstl, 2003; Bell and Zlokovic, 2009). Converging on this idea, the ethiopathogenetic role of a spectrum of chronic vascular disorders such as hypertension, hypercholesterolemia and type 2 diabetes has been proven to be present in the pathogenesis of AD (Kalaria, 2012).

The alterations in cerebrovascular function in aging and AD can be reflected by both chronic brain hypoperfusion and altered neurovascular coupling. Numerous clinical studies, based on evaluation of resting CBF in human subjects, unanimously recognize a negative correlation between global CBF and age (Krejza et al., 1999; Schultz et al., 1999; Fisher et al., 2013; Fabiani et al., 2014). Also, several lines of evidence support cerebral hypoperfusion as a preclinical condition in AD and one of the most accurate predictors for developing AD. Studies of brain function during behavioral tasks suggest age-related differences in activation, as well as differences between patients with AD and age-matched control subjects, revealing a close correlation with cognitive decline (Alsop et al., 2000; Ruitenberg et al., 2005; Xu et al., 2007). A significant observation is that the BOLD signal evaluated by fMRI during an associative encoding task is reduced in individuals carrying the APOE-ε 4 allele, a recognized risk factor for sporadic AD (Fleisher et al., 2009).

However, whereas the correlation between cerebrovascular changes and cognitive decline has been firmly established, the neurobiological link between the two is still poorly defined, as is the causality (Iadecola, 2004). Evidence for cerebrovascular changes resulting in neuronal damage, hastening neurodegeneration, is clear but it is also known that neurogenic factors can underlie cerebrovascular dysfunction. Amyloid β peptide (Aβ), the main component of the amyloid plaques found in AD, may play a major role in cerebrovascular impairment, as it is known to disrupt the physiological mechanisms regulating CBF. Elevations of Aβ levels in rodent models of AD are associated with lower resting CBF and impaired vasodilatory responses in cerebral circulation (Iadecola et al., 1999; Niwa et al., 2002), including neurovascular coupling (Niwa et al., 2000a). Additionally, Aβ peptides can impair endothelium-dependent relaxation and enhance vasoconstriction (Thomas et al., 1996; Niwa et al., 2000b, 2001) by either promoting oxidative stress in the vascular cells (Hamel et al., 2008) and/or inhibiting the production of neuronal-derived vasodilating messengers (Iadecola, 2004) such as ^•^NO (Venturini et al., 2002).

**Figure 1:**
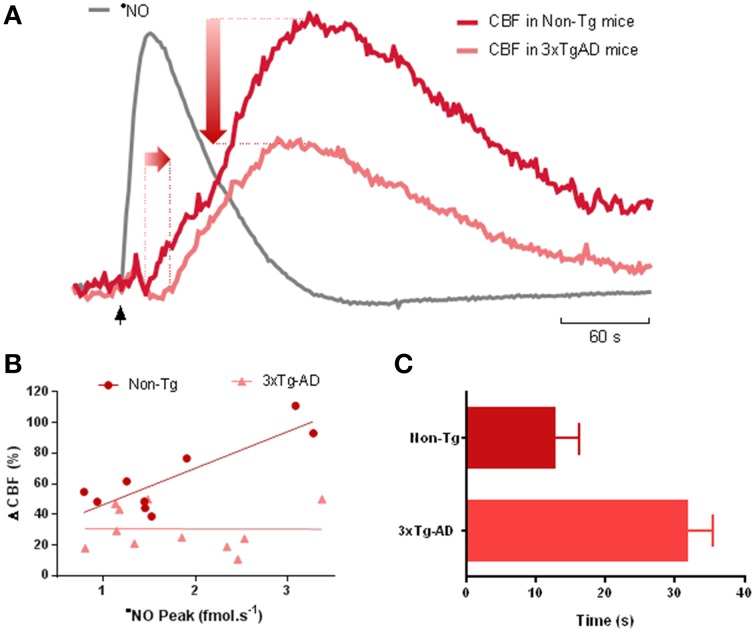
**Impairment of nitric oxide-dependent neurovascular coupling in AD. (A)** Representative recordings of the simultaneous measurements of ^•^NO concentration dynamics and CBF changes in the hippocampus of 12 months-old Non-Tg mice and 3xTg-AD mice in response to L-glutamate. L-glutamate (0.5 nmol, 1 s) was locally applied at time indicated by the black vertical arrow. The measurements were performed using ^•^NO selective microelectrodes and Laser Doppler flowmetry as previously described (Lourenço et al., 2014a). The temporal dynamic of ^•^NO is roughly identical in both strains and thus only a representative trace is presented (gray line). The CBF change coupled to the transient ^•^NO increase showed a temporal delay and decrease in amplitude in 3xTg-AD mice (light red line) as compared to Non-Tg mice (dark red line). **(B)** The linear relationship between ^•^NO peak amplitude and the amplitude CBF change observed in Non-Tg mice (*p* = 0.003) was lost in 3xTg-AD mice (*p* = 0.981). In the former, the linear regression showed an *R^2^ = 0.735* and a slope of 24% CBF/fmol s^−1^^•^NO. **(C)** Delay between the onset of ^•^NO transient and the onset of the CBF change in 12-months old 3xTg-AD mice and Non-Tg mice. Values represent the mean ± SEM (*p* = 0.001).

In an attempt to help clarify this controversy, we have observed age-dependent impairment of ^•^NO-dependent neurovascular coupling both in rodent models of AD and aging. Furthermore, we found that this dysfunction appears to be primarily of cerebrovascular rather than neuronal origin. These studies were carried out in a triple transgenic mouse model of AD (3xTg-AD mice) and in Fisher 344 rats (widely used in brain aging studies). By simultaneously measuring ^•^NO and CBF in the hippocampus, we observed that glutamate-evoked increase in CBF was diminished during aging and AD, despite the fact that ^•^NO signaling remains almost unaltered. The effect of aging/AD on ^•^NO-dependent CBF changes is summarized in Figure 1. Data obtained in 3xTg-AD mice revealed a shift in the CBF changes coupled to the ^•^NO temporal dynamic elicited by glutamatergic activation. This is reflected by the increased delay on the onset of CBF increase (Figure 1C), as well as by the decrease in the amplitude of CBF change. The later leads to the abolishment of the correlation between ^•^NO and CBF observed in control animals (Figure 1B). Similar observations were obtained during aging in F344 rats, with CBF changes declining 39% from 6 to 12 months, and a further 36% from 12 to 23 months, with no significant decrease in ^•^NO signaling (unpublished data). Of note, the deterioration of the neurovascular coupling in both cases preceded an obvious impairment in cognitive function as accessed by behavior tests.

Overall, these results strengthen the notion of cerebrovascular dysfunction as a fundamental process underlying AD pathophysiology and brain aging. That is, while neuronal ^•^NO signaling remains functional it is not conveniently transduced into vasodilation at blood vessels. Amongst the potential causes, changes of the redox environment in blood vessels may result in the quenching of ^•^NO, impeding it's binding to sGC. In fact, previous reports show that vascular oxidative stress may have serious implications in cerebrovascular function (reviewed in Hamel et al., 2008).

A deep understanding of the underlying mechanisms by which altered cerebrovascular function influences AD neuropathology has not yet been achieved. Nevertheless, in accordance with the hypothesis prompted by the vascular-driven theory of AD, cerebrovascular dysfunction and consequent hypoperfusion is suggested to be linked to (1) enhanced β-secretase protein expression, which potentiates Aβ overproduction and altered phosphorylated tau (Velliquette et al., 2005; Koike et al., 2010); (2) faulty clearance of Aβ peptides, favoring accumulation in the brain (Girouard and Iadecola, 2006) and (3) reduced supply of metabolic substrates and neurometabolic dysfunction.

## Altered neurometabolism in brain aging and Alzheimer's disease

Brain aging is accompanied by widespread metabolic alterations associated with cognitive decline that connect with the so called “metabolic syndrome” (Barzilai et al., 2012). Components of metabolic syndrome such as insulin resistance or hypercholesterolemia are predictors of accelerated cognitive decline and dementia, particularly AD (Haralampos et al., 2008). The pathways linking these metabolic alterations and the decay of cognitive function are still poorly understood, but mitochondrial dysfunction has been identified as a link between the two. Besides changes in neurotransmission processes (decrease in glutamate and GABA), brain aging and AD are associated with perturbations of primary energy metabolism (use of glucose and lactate) as well as in the turnover of lipid membranes (Castegna et al., 2004; Mohmmad Abdul and Butterfield, 2005; Bader Lange et al., 2008; Duarte et al., 2014). Mitochondria play an essential role in cellular respiration and are responsible not only for the production of ATP, but also for Ca^2+^ buffering, production and removal of reactive oxygen species, as well as of signaling molecules that regulate cell cycle, proliferation and apoptosis (for review Yin et al., 2014). The brain's consumption of glucose is primarily driven by the constant need to maintain ionic gradients in pre- and post-synaptic compartments in order to sustain excitability, as well as to maintain transmembrane lipid asymmetries (Harris et al., 2012). Considering the high energetic demand of this organ and it's excitability requirements, deregulation of optimal mitochondrial performance can significantly impact neurometabolism and function.

During the past decades, intensive research has shown that brain energy metabolism in impaired during the progression of AD (de Leon et al., 1983; Mosconi et al., 2005; Reiman et al., 2005; Scholl et al., 2011; Yin et al., 2014). Amyloidosis, or more specifically plaque pathology (Gearing et al., 1995), although present in roughly 90% of AD patients, is not in itself sufficient to account for the disease and many authors have reported that amyloid load may not necessarily correlate with dementia (Gearing et al., 1995; Hsia et al., 1999; Mucke et al., 2000; Swerdlow et al., 2014). As the amyloid cascade hypothesis of AD has evolved over the years, the concept of plaque as the causal factor of disease has given way to the notion that soluble forms of Aβ oligomers are in fact the toxic moiety (Lesne and Kotilinek, 2005; Walsh et al., 2005; Lesne et al., 2006). These oligomers have been shown to compromise the function of organelles such as the mitochondria.

Thus, the concept that mitochondrial dysfunction is a key contributor to the onset and progression of AD has become firmly consolidated and the “mitochondrial cascade hypothesis” has gained significant ground, especially with regards to late-onset AD (Chaturvedi and Flint Beal, 2013; Swerdlow et al., 2014). This hypothesis is sustained not only on the fact that both Aβ and hyperphosphorylated tau are capable of altering mitochondrial function, but also takes into account the major risk factor for sporadic AD—age. One important consequence of biological aging is the accumulation of somatic mtDNA mutations, which contribute to physiological decline and neurodegenerative disease (Lin et al., 2002). The impact of these point mutations on each individual as they age will be influenced by inherited and environmental factors (Wallace, 1992; Swerdlow et al., 2014).

Studies in both animal models and AD patients have shown that Aβ is capable of inhibiting the complexes of the mitochondrial respiratory chain, as well as the TCA cycle enzyme α-ketogluterate dehydrogenase (Casley et al., 2002; Manczak et al., 2006). Impairment of oxidative phosphorylation (OxPhos) is mainly due to the inhibition or decreased activity of CcO, which, along with ATP synthase, is known to be oxidized in the brains of AD patients (Kish et al., 1992; Mutisya et al., 1994; Maurer et al., 2000). Furthermore, mitochondrial dysfunction promotes tangle formation in AD by contributing to tau phosphorylation (Melov et al., 2007).

**Figure 2:**
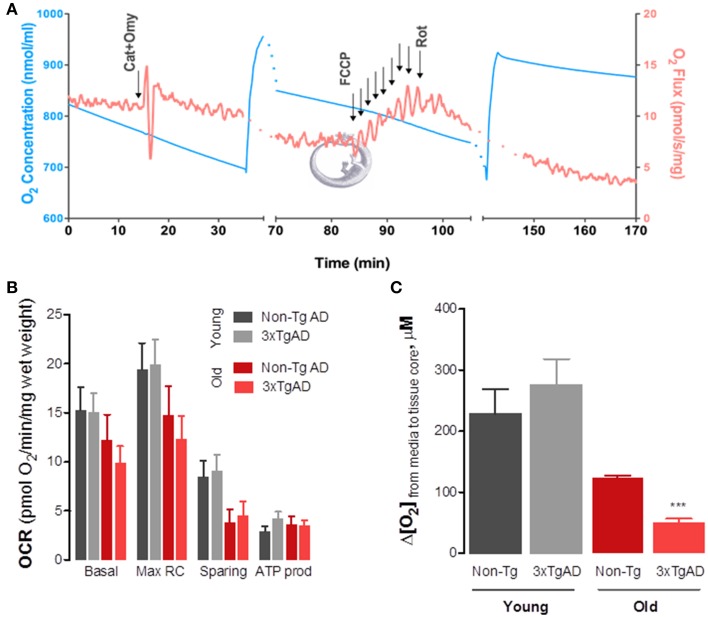
**Changes in mitochondrial oxidative phosphorylation in intact hippocampal slices from 3xTg-AD mice and Non-Tg mice show significant effect of aging on basal and maximal respiratory rates as well as sparing capacity**. We developed a protocol that enabled us to evaluate OxPhos in intact hippocampal slices obtained from young and old-aged mice. Using a high-resolution respirometer (Oxygraph-2K, by Oroboros Instruments, Austria) we determined O_2_ consumption rates (OCR) or O_2_ flux (red line in **A**). Due to high O_2_ requirement of hippocampal slices, experiments were performed at high [O_2_] and chambers were re-oxygenated throughout the experiment (blue line in **A**). Basal OCR was obtained in BSA-supplemented media containing 10 mM glucose+pyruvate. Carboxyatractyloside and oligomycin (CAT+Omy; 12.5 μM and 20 μg/mL) were then added to determine OCR not dependent on ATP production (leak). Maximal respiratory rate was achieved by titration with FCCP (20 μM), following which non-mitochondrial respiration was determined by adding rotenone (Rot, 2.2 μM). From each recording we determined the OCR values presented in **(B)**. Two-Way ANOVA analysis revealed a significant effect of age on both maximal (*F* = 4.69; *P* = 0.0368) and sparing capacity (*F* = 7.39; ^***^p = 0.01). In **(C)** one can observe that the drop in [O_2_] from the medium bathing the hippocampal slice (aCSF bubbled with 95%O_2_/5%CO_2_ gas mixture, at 32°C) is significantly decreased in old-aged 3xTg-AD, further supporting respirometry data showing decrease in basal metabolic rate. This drop was determined electrochemically using carbon fiber microelectrodes held at −0, 8 V vs. Ag/AgCl and lowered from the perfusion media into the slice core gradually (see Ledo et al., 2005 for detailed description).

Using high resolution respirometry for evaluation of O_2_ consumption rates (OCR) in intact hippocampal slices obtained from 3xTg-AD mice and age-matched controls (Figure 2A) we have observed an age-dependent impairment of OxPhos in old-aged animals which tends to be more evident in old-aged 3xTg-AD (Figure 2B). Both basal and maximal O_2_ consumption rates decrease as a function of age in both genotypes. More relevantly, the sparing capacity has diminished in both old-age groups, implying a lower capacity to respond to energy-demanding situations (such as increased excitability) with adequate increase OxPhos to supply ATP. Decrease in basal tissue OCR was further revealed when we determined the drop in [O_2_] from the surrounding media to the tissue core (Figure 2C), which was significantly smaller in old-age 3TgAD animals. This drop in [O_2_] is expected to positively correlate with the metabolic activity state of the tissue. Of note is the fact that this 3xTg-AD model, although expressing a progressive AD phenotype as expected to occur in humans (Oddo et al., 2003), is a model of familiar AD and not sporadic AD. As such, it is not surprising to us to find that the major contributor to changes in OxPhos is actually age and not genotype. Sporadic AD is not associated with deterministic gene mutations, although some genetic influences have been identified, such as the APOE-ε 4 variant (Corder et al., 1993; Swerdlow, 2007).

Hypometabolism in AD is most likely more than a mere consequence of the cellular and functional degeneration (decreased brain function would obviously require less energy substrate supply)—recent observations suggest that optimal glucose utilization is impaired in early asymptomatic stages of the disease and may contribute or precipitate AD neuropathology. Data collected from both clinical and animal model research strongly suggest that significant decrease in brain metabolism occurs well before any clinical manifestation of AD, namely measurable cognitive decline (for review Petrella et al., 2003).

Changes in the concentration dynamics of ^•^NO in the brain may contribute to altered neurometabolism in aging and AD. In structures of the CNS intimately linked to memory and learning, such as the hippocampus, ^•^NO is produced by neurons upon activation of glutamatergic synapses (Ledo et al., 2005, 2015; Lourenço et al., 2011, 2014b). Besides its obvious role as a neuromodulator, acting namely as a retrograde messenger in plasticity phenomena associated with memory and learning (Prast and Philippu, 2001), ^•^NO has been looked upon as a master regulator of neurometabolism, as discussed above. Alongside or as a consequence of inhibition of the mitochondrial electron transporting chain (Antunes and Cadenas, 2007), ^•^NO has also been shown to boost glycolytic rate and glucose uptake (reviewed in Almeida et al., 2005). One can hypothesize that changes in either ^•^NO concentration dynamics or cellular redox environment toward a more oxidative status (which promotes production of reactive oxygen and nitrogen species), not only detours ^•^NO from its physiological role but also precipitates the production of highly oxidative and nitrosative sprecies such as peroxynitrite and dinitrogen trioxide (Heinrich et al., 2013). In line with this hypothesis, increase in tyrosine nitration is observed in the brains of AD patients when compared to healthy age-matched individuals, indicating changes in ^•^NO bioactivity (Fernandez-Vizarra et al., 2004).

Using hippocampal slices to measure both NMDA-evoked ^•^NO production and changes in O_2_ profiles in the CA1 subregion, we have previously shown that ^•^NO may act as a modulator of neurometabolic rate upon stimulation of glutamatergic transmission (Ledo et al., 2010). In hippocampal slices obtained from old-age 3xTg-AD mice, and looking specifically at the CA1 pyramidal layer, we observe decreased ^•^NO upon activation of NMDA receptor (unpublished data), which most likely results from changes in ^•^NO bioavailability due to its rapid reaction with species such as superoxide radical to produce peroxynitrite. As a consequence, we also observed that the tight coupling between neuronal-^•^NO and inhibition of O_2_ consumption as shown in healthy subjects is lost, suggesting that ^•^NO is no longer capable or available to act as the master regulator of neurometabolic coupling.

## Conclusion

An increasing amount of evidence supports the notion that Alzheimer's disease is a multifaceted pathology that goes far beyond the amyloid pathology. As a major risk factor for AD, aging shares many common features associated with functional decline, namely neurovascular and neurometabolic alterations. Although the causal role of these changes in AD pathology remains controversial, it seems increasingly certain that they significantly impact the progression of neuronal dysfunction. Furthermore, the imbalance in the regulation of the neurovascular and neurometabolic coupling, resulting from cerebrovascular dysfunction, appear to be precocious events in neurodegeneration and brain aging. This shift in paradigm and the role of vascular redox status of brain microcirculation may be crucial for development of adequate therapeutically strategies that hamper cognition defects and neurodegeneration.

### Conflict of interest statement

The Review Editor Paula I. Moreira declares that, despite being affiliated to the same institution as authors Cátia F. Lourenço, Ana Ledo, Cândida Dias, Rui M. Barbosa and João Laranjinha, the review process was handled objectively and no conflict of interest exists. The authors declare that the research was conducted in the absence of any commercial or financial relationships that could be construed as a potential conflict of interest.

## References

[B1] Almeida A. Cidad P. Delgado-Esteban M. Fernandez E. Garcia-Nogales P. Bolanos J. P. (2005). Inhibition of mitochondrial respiration by nitric oxide: its role in glucose metabolism and neuroprotection. J. Neurosci. Res. 79, 166–171. 10.1002/jnr.20281 15573411

[B2] Alsop D. C. Detre J. A. Grossman M. (2000). Assessment of cerebral blood flow in Alzheimer's disease by spin-labeled magnetic resonance imaging. Ann. Neurol. 47, 93–100. 10.1002/1531-8249(200001)47:1<93::AID-ANA15>3.0.CO;2-8 10632106

[B3] Antunes F. Cadenas E. (2007). The mechanism of cytochrome C oxidase inhibition by nitric oxide. Front. Biosci. 12:2118. 10.2741/2118 17127353

[B4] Attwell D. Buchan A. M. Charpak S. Lauritzen M. Macvicar B. A. Newman E. A. (2010). Glial and neuronal control of brain blood flow. Nature 468, 232–43. 10.1038/nature09613 21068832 PMC3206737

[B5] Bader Lange M. L. Cenini G. Piroddi M. Abdul H. M. Sultana R. Galli F. (2008). Loss of phospholipid asymmetry and elevated brain apoptotic protein levels in subjects with amnestic mild cognitive impairment and Alzheimer disease. Neurobiol. Dis. 29, 456–464. 10.1016/j.nbd.2007.11.004 18077176 PMC2292396

[B6] Barzilai N. Huffman D. M. Muzumdar R. H. Bartke A. (2012). The critical role of metabolic pathways in aging. Diabetes 61, 1315–1322. 10.2337/db11-1300 22618766 PMC3357299

[B7] Bell R. D. Zlokovic B. V. (2009). Neurovascular mechanisms and blood-brain barrier disorder in Alzheimer's disease. Acta Neuropathol. 118, 103–113. 10.1007/s00401-009-0522-3 19319544 PMC2853006

[B8] Bolanos J. P. Delgado-Esteban M. Herrero-Mendez A. Fernandez-Fernandez S. Almeida A. (2008). Regulation of glycolysis and pentose-phosphate pathway by nitric oxide: impact on neuronal survival. Biochim. Biophys. Acta 1777, 789–793. 10.1016/j.bbabio.2008.05.179 18455501

[B9] Brown G. C. (2007). Nitric oxide and mitochondria. Front. Biosci. 12:2122 10.2741/2122 17127357

[B10] Cadenas E. (2004). Mitochondrial free radical production and cell signaling. Mol. Aspects Med. 25, 17–26. 10.1016/j.mam.2004.02.005 15051313

[B11] Casley C. S. Land J. M. Sharpe M. A. Clark J. B. Duchen M. R. Canevari L. (2002). Beta-amyloid fragment 25-35 causes mitochondrial dysfunction in primary cortical neurons. Neurobiol. Dis. 10, 258–267. 10.1006/nbdi.2002.0516 12270688

[B12] Castegna A. Lauderback C. M. Mohmmad-Abdul H. Butterfield D. A. (2004). Modulation of phospholipid asymmetry in synaptosomal membranes by the lipid peroxidation products, 4-hydroxynonenal and acrolein: implications for Alzheimer's disease. Brain Res. 1004, 193–197. 10.1016/j.brainres.2004.01.036 15033435

[B13] Chaturvedi R. K. Flint Beal M. (2013). Mitochondrial diseases of the brain. Free Radic. Biol. Med. 63, 1–29. 10.1016/j.freeradbiomed.2013.03.018 23567191

[B14] Christopherson K. S. Hillier B. J. Lim W. A. Bredt D. S. (1999). PSD-95 assembles a ternary complex with the N-methyl-D-aspartic acid receptor and a bivalent neuronal NO synthase PDZ domain. J. Biol. Chem. 274, 27467–27473. 10.1074/jbc.274.39.27467 10488080

[B15] Corder E. H. Saunders A. M. Strittmatter W. J. Schmechel D. E. Gaskell P. C. Small G. W.. (1993). Gene dose of apolipoprotein E type 4 allele and the risk of Alzheimer's disease in late onset families. Science 261, 921–923. 10.1126/science.8346443 8346443

[B16] de la Torre J. C. (2012). Cerebral hemodynamics and vascular risk factors: setting the stage for Alzheimer's disease. J. Alzheimers Dis. 32, 553–567. 10.3233/JAD-2012-120793 22842871

[B17] de Leon M. J. Ferris S. H. George A. E. Christman D. R. Fowler J. S. Gentes C.. (1983). Positron emission tomographic studies of aging and Alzheimer disease. AJNR Am. J. Neuroradiol. 4, 568–571. 6410799 PMC8334899

[B18] Duarte J. M. Schuck P. F. Wenk G. L. Ferreira G. C. (2014). Metabolic disturbances in diseases with neurological involvement. Aging Dis. 5, 238–255. 10.14336/AD.2014.0500238 25110608 PMC4113514

[B19] Fabiani M. Gordon B. A. Maclin E. L. Pearson M. A. Brumback-Peltz C. R. Low K. A.. (2014). Neurovascular coupling in normal aging: a combined optical, ERP and fMRI study. Neuroimage 85(Pt 1), 592–607. 10.1016/j.neuroimage.2013.04.113 23664952 PMC3791333

[B20] Fernandez-Vizarra P. Fernandez A. P. Castro-Blanco S. Encinas J. M. Serrano J. Bentura M. L.. (2004). Expression of nitric oxide system in clinically evaluated cases of Alzheimer's disease. Neurobiol. Dis. 15, 287–305. 10.1016/j.nbd.2003.10.010 15006699

[B21] Fisher J. P. Hartwich D. Seifert T. Olesen N. D. McNulty C. L. Nielsen H. B.. (2013). Cerebral perfusion, oxygenation and metabolism during exercise in young and elderly individuals. J. Physiol. 591(Pt 7), 1859–1870. 10.1113/jphysiol.2012.244905 23230234 PMC3624856

[B22] Fleisher A. S. Podraza K. M. Bangen K. J. Taylor C. Sherzai A. Sidhar K.. (2009). Cerebral perfusion and oxygenation differences in Alzheimer's disease risk. Neurobiol. Aging 30, 1737–1748. 10.1016/j.neurobiolaging.2008.01.012 18325636 PMC2746874

[B23] Gearing M. Mirra S. S. Hedreen J. C. Sumi S. M. Hansen L. A. Heyman A. (1995). The Consortium to Establish a Registry for Alzheimer's Disease (CERAD). Part X. Neuropathology confirmation of the clinical diagnosis of Alzheimer's disease. Neurology 45(3 Pt 1), 461–466. 10.1212/WNL.45.3.461 7898697

[B24] Girouard H. Iadecola C. (2006). Neurovascular coupling in the normal brain and in hypertension, stroke, and Alzheimer disease. J. Appl. Physiol. 100, 328–335. 10.1152/japplphysiol.00966.2005 16357086

[B25] Giulivi C. (2003). Characterization and function of mitochondrial nitric-oxide synthase. Free Radic. Biol. Med. 34, 397–408. 10.1016/S0891-5849(02)01298-4 12566065

[B26] Gordon G. R. Choi H. B. Rungta R. L. Ellis-Davies G. C. MacVicar B. A. (2008). Brain metabolism dictates the polarity of astrocyte control over arterioles. Nature 456, 745–79. 10.1038/nature07525 18971930 PMC4097022

[B27] Guix F. X. Uribesalgo I. Coma M. Munoz F. J. (2005). The physiology and pathophysiology of nitric oxide in the brain. Prog. Neurobiol. 76, 126–152. 10.1016/j.pneurobio.2005.06.001 16115721

[B28] Hamel E. Nicolakakis N. Aboulkassim T. Ongali B. Tong X. K. (2008). Oxidative stress and cerebrovascular dysfunction in mouse models of Alzheimer's disease. Exp. Physiol. 93, 116–120. 10.1113/expphysiol.2007.038729 17911359

[B29] Haralampos J. M. Matilda F. Sotirios G. (2008). Metabolic syndrome and Alzheimer's disease: a link to a vascular hypothesis? CNS Spectr. 13, 606–613. 10.1017/S1092852900016886 18622365

[B30] Harris J. J. Jolivet R. Attwell D. (2012). Synaptic energy use and supply. Neuron 75, 762–777. 10.1016/j.neuron.2012.08.019 22958818

[B31] Heinrich T. A. da Silva R. S. Miranda K. M. Switzer C. H. Wink D. A. Fukuto J. M. (2013). Biological nitric oxide signaling: chemistry and terminology (NO chemical biology and terminology). Br. J. Pharmacol. 169, 1417–1429. 10.1111/bph.12217 23617570 PMC3724101

[B32] Hsia A. Y. Masliah E. McConlogue L. Yu G. Q. Tatsuno G. Hu K.. (1999). Plaque-independent disruption of neural circuits in Alzheimer's disease mouse models. Proc. Natl. Acad. Sci. U.S.A. 96, 3228–3233. 10.1073/pnas.96.6.3228 10077666 PMC15924

[B33] Iadecola C. (1993). Regulation of the cerebral microcirculation during neural activity: is nitric oxide the missing link? Trends Neurosci. 16, 206–214. 10.1016/0166-2236(93)90156-G 7688160

[B34] Iadecola C. (2004). Neurovascular regulation in the normal brain and in Alzheimer's disease. Nat. Rev. Neurosci. 5, 347–360. 10.1038/nrn1387 15100718

[B35] Iadecola C. Zhang F. Niwa K. Eckman C. Turner S. K. Fischer E.. (1999). SOD1 rescues cerebral endothelial dysfunction in mice overexpressing amyloid precursor protein. Nat. Neurosci. 2, 157–161. 10.1038/5715 10195200

[B36] Jellinger K. A. Mitter-Ferstl E. (2003). The impact of cerebrovascular lesions in Alzheimer disease–a comparative autopsy study. J. Neurol. 250, 1050–1055. 10.1007/s00415-003-0142-0 14504965

[B37] Kalaria R. N. (2012). Cerebrovascular disease and mechanisms of cognitive impairment: evidence from clinicopathological studies in humans. Stroke 43, 2526–2534. 10.1161/strokeaha.112.655803 22879100

[B38] Kealy J. Bennett R. Lowry J. P. (2013). Simultaneous recording of hippocampal oxygen and glucose in real time using constant potential amperometry in the freely-moving rat. J. Neurosci. Methods 215, 110–120. 10.1016/j.jneumeth.2013.02.016 23499196

[B39] Kish S. J. Bergeron C. Rajput A. Dozic S. Mastrogiacomo F. Chang L. J.. (1992). Brain cytochrome oxidase in Alzheimer's disease. J. Neurochem. 59, 776–779. 10.1111/j.1471-4159.1992.tb09439.x 1321237

[B40] Koike M. A. Green K. N. Blurton-Jones M. Laferla F. M. (2010). Oligemic hypoperfusion differentially affects tau and amyloid-{beta}. Am. J. Pathol. 177, 300–310. 10.2353/ajpath.2010.090750 20472896 PMC2893673

[B41] Krejza J. Mariak Z. Walecki J. Szydlik P. Lewko J. Ustymowicz A. (1999). Transcranial color Doppler sonography of basal cerebral arteries in 182 healthy subjects: age and sex variability and normal reference values for blood flow parameters. AJR Am. J. Roentgenol. 172, 213–218. 10.2214/ajr.172.1.9888770 9888770

[B42] Ledo A. Barbosa R. Cadenas E. Laranjinha J. (2010). Dynamic and interacting profiles of ^*^NO and O2 in rat hippocampal slices. Free Radic. Biol. Med. 48, 1044–1050. 10.1016/j.freeradbiomed.2010.01.024 20100565 PMC2839026

[B43] Ledo A. Barbosa R. Gerhardtt G. Cadenas E. Laranjinha J. (2005). Concentration dynamics of nitric oxide in rat hippocampal subregions evoked by stimulation of the NMDA glutamate receptor. Proc. Natl. Acad. Sci. U.S.A. 102, 17483–17488. 10.1073/pnas.0503624102 16293699 PMC1297656

[B44] Ledo A. Lourenço C. F. Caetano M. Barbosa R. M. Laranjinha J. (2015). Age-associated changes of nitric oxide concentration dynamics in the central nervous system of fisher 344 rats. Cell. Mol. Neurobiol. 35, 33–44. 10.1007/s10571-014-0115-0 25274046 PMC11488054

[B45] Lesne S. Koh M. T. Kotilinek L. Kayed R. Glabe C. G. Yang A.. (2006). A specific amyloid-beta protein assembly in the brain impairs memory. Nature 440, 352–357. 10.1038/nature04533 16541076

[B46] Lesne S. Kotilinek L. (2005). Amyloid plaques and amyloid-beta oligomers: an ongoing debate. J. Neurosci. 25, 9319–9320. 10.1523/JNEUROSCI.3246-05.2005 16221839 PMC6725700

[B47] Lin M. T. Simon D. K. Ahn C. H. Kim L. M. Beal M. F. (2002). High aggregate burden of somatic mtDNA point mutations in aging and Alzheimer's disease brain. Hum. Mol. Genet. 11, 133–145. 10.1093/hmg/11.2.133 11809722

[B48] Lourenço C. F. Ferreira N. R. Santos R. M. Lukacova N. Barbosa R. M. Laranjinha J. (2014b). The pattern of glutamate-induced nitric oxide dynamics *in vivo* and its correlation with nNOS expression in rat hippocampus, cerebral cortex and striatum. Brain Res. 1554, 1–11. 10.1016/j.brainres.2014.01.030 24495843

[B49] Lourenço C. F. Santos R. Barbosa R. M. Gerhardt G. Cadenas E. Laranjinha J. (2011). *In vivo* modulation of nitric oxide concentration dynamics upon glutamatergic neuronal activation in the hippocampus. Hippocampus 21, 622–630. 10.1002/hipo.20774 20169537

[B50] Lourenço C. F. Santos R. M. Barbosa R. M. Cadenas E. Radi R. Laranjinha J. (2014a). Neurovascular coupling in hippocampus is mediated via diffusion by neuronal-derived nitric oxide. Free Radic. Biol. Med. 73, 421–49. 10.1016/j.freeradbiomed.2014.05.021 24887095

[B51] Manczak M. Anekonda T. S. Henson E. Park B. S. Quinn J. Reddy P. H. (2006). Mitochondria are a direct site of A beta accumulation in Alzheimer's disease neurons: implications for free radical generation and oxidative damage in disease progression. Hum. Mol. Genet. 15, 1437–1449. 10.1093/hmg/ddl066 16551656

[B52] Maurer I. Zierz S. Möller H. J. (2000). A selective defect of cytochrome c oxidase is present in brain of Alzheimer disease patients. Neurobiol. Aging 21, 455–462. 10.1016/S0197-4580(00)00112-3 10858595

[B53] Melov S. Adlard P. A. Morten K. Johnson F. Golden T. R. Hinerfeld D.. (2007). Mitochondrial oxidative stress causes hyperphosphorylation of tau. PLoS ONE 2:e536 10.1371/journal.pone.0000536 17579710 PMC1888726

[B54] Mohmmad Abdul H. Butterfield D. A. (2005). Protection against amyloid beta-peptide (1-42)-induced loss of phospholipid asymmetry in synaptosomal membranes by tricyclodecan-9-xanthogenate (D609) and ferulic acid ethyl ester: implications for Alzheimer's disease. Biochim. Biophys. Acta 1741, 140–148. 10.1016/j.bbadis.2004.12.002 15955457

[B55] Moncada S. Bolaños J. P. (2006). Nitric oxide, cell bioenergetics and neurodegeneration. J. Neurochem. 97, 1676–189. 10.1111/j.1471-4159.2006.03988.x 16805776

[B56] Mosconi L. Tsui W. H. De Santi S. Li J. Rusinek H. Convit A.. (2005). Reduced hippocampal metabolism in MCI and AD: automated FDG-PET image analysis. Neurology 64, 1860–1867. 10.1212/01.WNL.0000163856.13524.08 15955934

[B57] Mucke L. Yu G. Q. McConlogue L. Rockenstein E. M. Abraham C. R. Masliah E. (2000). Astroglial expression of human alpha(1)-antichymotrypsin enhances alzheimer-like pathology in amyloid protein precursor transgenic mice. Am. J. Pathol. 157, 2003–2010. 10.1016/S0002-9440(10)64839-0 11106573 PMC1885780

[B58] Mutisya E. M. Bowling A. C. Beal M. F. (1994). Cortical cytochrome oxidase activity is reduced in Alzheimer's disease. J. Neurochem. 63, 2179–2184. 10.1046/j.1471-4159.1994.63062179.x 7964738

[B59] Niwa K. Carlson G. A. Iadecola C. (2000b). Exogenous A beta1-40 reproduces cerebrovascular alterations resulting from amyloid precursor protein overexpression in mice. J. Cereb. Blood Flow Metab. 20, 1659–1668. 10.1097/00004647-200012000-00005 11129782

[B60] Niwa K. Kazama K. Younkin S. G. Carlson G. A. Iadecola C. (2002). Alterations in cerebral blood flow and glucose utilization in mice overexpressing the amyloid precursor protein. Neurobiol. Dis. 9, 61–68. 10.1006/nbdi.2001.0460 11848685

[B61] Niwa K. Porter V. A. Kazama K. Cornfield D. Carlson G. A. Iadecola C. (2001). A beta-peptides enhance vasoconstriction in cerebral circulation. Am. J. Physiol. Heart Circ. Physiol. 281, H2417–H2424 11709407 10.1152/ajpheart.2001.281.6.H2417

[B62] Niwa K. Younkin L. Ebeling C. Turner S. K. Westaway D. Younkin S.. (2000a). Abeta 1-40-related reduction in functional hyperemia in mouse neocortex during somatosensory activation. Proc. Natl. Acad. Sci. U.S.A. 97, 9735–9740. 10.1073/pnas.97.17.9735 10944232 PMC16934

[B63] Oddo S. Caccamo A. Shepherd J. D. Murphy M. P. Golde T. E. Kayed R.. (2003). Triple-transgenic model of Alzheimer's disease with plaques and tangles: intracellular Abeta and synaptic dysfunction. Neuron 39, 409–421. 10.1016/S0896-6273(03)00434-3 12895417

[B64] Palamalai V. Miyagi M. (2010). Mechanism of glyceraldehyde-3-phosphate dehydrogenase inactivation by tyrosine nitration. Protein Sci. 19, 255–62. 10.1002/pro.311 20014444 PMC2865723

[B65] Petrella J. R. Coleman R. E. Doraiswamy P. M. (2003). Neuroimaging and early diagnosis of Alzheimer disease: a look to the future. Radiology 226, 315–336. 10.1148/radiol.2262011600 12563122

[B66] Prast H. Philippu A. (2001). Nitric oxide as modulator of neuronal function. Prog. Neurobiol. 64, 51–68. 10.1016/S0301-0082(00)00044-7 11250062

[B67] Rancillac A. Rossier J. Guille M. Tong X. K. Geoffroy H. Amatore C.. (2006). Glutamatergic control of microvascular tone by distinct GABA neurons in the cerebellum. J. Neurosci. 26, 6997–7006. 10.1523/jneurosci.5515-05.2006 16807329 PMC6673912

[B68] Reiman E. M. Chen K. Alexander G. E. Caselli R. J. Bandy D. Osborne D.. (2005). Correlations between apolipoprotein E epsilon4 gene dose and brain-imaging measurements of regional hypometabolism. Proc. Natl. Acad. Sci. U.S.A. 102, 8299–8302. 10.1073/pnas.0500579102 15932949 PMC1149416

[B69] Rossignol R. Faustin B. Rocher C. Malgat M. Mazat J. P. Letellier T. (2003). Mitochondrial threshold effects. Biochem. J. 370(Pt 3), 751–62. 10.1042/bj20021594 12467494 PMC1223225

[B70] Ruitenberg A. den Heijer T. Bakker S. L. van Swieten J. C. Koudstaal P. J. Hofman A.. (2005). Cerebral hypoperfusion and clinical onset of dementia: the Rotterdam Study. Ann. Neurol. 57, 789–794. 10.1002/ana.20493 15929050

[B71] Scholl M. Almkvist O. Axelman K. Stefanova E. Wall A. Westman E.. (2011). Glucose metabolism and PIB binding in carriers of a His163Tyr presenilin 1 mutation. Neurobiol. Aging 32, 1388–1399. 10.1016/j.neurobiolaging.2009.08.016 19796846

[B72] Schultz S. K. O'Leary D. S. Boles Ponto L. L. Watkins G. L. Hichwa R. D. Andreasen N. C. (1999). Age-related changes in regional cerebral blood flow among young to mid-life adults. Neuroreport 10, 2493–2496. 10.1097/00001756-199908200-00011 10574358

[B73] Shulman R. G. Rothman D. L. (1998). Interpreting functional imaging studies in terms of neurotransmitter cycling. Proc. Natl. Acad. Sci. U.S.A. 95, 11993–11998. 10.1073/pnas.95.20.11993 9751778 PMC21753

[B74] Swerdlow R. H. (2007). Is aging part of Alzheimer's disease, or is Alzheimer's disease part of aging? Neurobiol. Aging 28, 1465–1480. 10.1016/j.neurobiolaging.2006.06.021 16876913

[B75] Swerdlow R. H. Burns J. M. Khan S. M. (2014). The Alzheimer's disease mitochondrial cascade hypothesis: progress and perspectives. Biochim. Biophys. Acta 1842, 1219–1231. 10.1016/j.bbadis.2013.09.010 24071439 PMC3962811

[B76] Thomas T. Thomas G. McLendon C. Sutton T. Mullan M. (1996). beta-Amyloid-mediated vasoactivity and vascular endothelial damage. Nature 380, 168–171. 10.1038/380168a0 8600393

[B77] Velliquette R. A. O'Connor T. Vassar R. (2005). Energy inhibition elevates beta-secretase levels and activity and is potentially amyloidogenic in APP transgenic mice: possible early events in Alzheimer's disease pathogenesis. J. Neurosci. 25, 10874–10883. 10.1523/JNEUROSCI.2350-05.2005 16306400 PMC6725876

[B78] Venturini G. Colasanti M. Persichini T. Fioravanti E. Ascenzi P. Palomba L.. (2002). Beta-amyloid inhibits NOS activity by subtracting NADPH availability. FASEB J. 16, 1970–1972. 10.1096/fj.02-0186fje 12397094

[B79] Victor V. M. Nunez C. D'Ocon P. Taylor C. T. Esplugues J. V. Moncada S. (2009). Regulation of oxygen distribution in tissues by endothelial nitric oxide. Circ. Res. 104, 1178–1183. 10.1161/circresaha.109.197228 19407240

[B80] Wallace D. C. (1992). Mitochondrial genetics: a paradigm for aging and degenerative diseases? Science 256, 628–632. 10.1126/science.1533953 1533953

[B81] Walsh D. M. Klyubin I. Shankar G. M. Townsend M. Fadeeva J. V. Betts V.. (2005). The role of cell-derived oligomers of Abeta in Alzheimer's disease and avenues for therapeutic intervention. Biochem. Soc. Trans. 33(Pt 5), 1087–1090. 10.1042/BST20051087 16246051

[B82] Winkler S. R. Luer M. S. (1998). Antiepileptic drug review: part 1. Surg. Neurol. 49, 449–452. 10.1016/S0090-3019(97)00223-1 9537667

[B83] Xu G. Antuono P. G. Jones J. Xu Y. Wu G. Ward D.. (2007). Perfusion fMRI detects deficits in regional CBF during memory-encoding tasks in MCI subjects. Neurology 69, 1650–1656. 10.1212/01.wnl.0000296941.06685.22 17954780

[B84] Yin F. Boveris A. Cadenas E. (2014). Mitochondrial energy metabolism and redox signaling in brain aging and neurodegeneration. Antioxid. Redox Signal. 20, 353–371. 10.1089/ars.2012.4774 22793257 PMC3887431

[B85] Zlokovic B. V. (2011). Neurovascular pathways to neurodegeneration in Alzheimer's disease and other disorders. Nat. Rev. Neurosci. 12, 723–738. 10.1038/nrn3114 22048062 PMC4036520

